# Adherent Perinephric Fat and Positive Surgical Margin in Laparoscopic Partial Nephrectomy: A Technique-Conditional Association Concentrated in Enucleation

**DOI:** 10.3390/jcm15176884

**Published:** 2026-09-05

**Authors:** Abdurrahman İnkaya, Hasan Samet Güngör, Ahmet Şanlı, Mehmet Sencer Sözen, Mehmet Umut Evci, Ali Kumcu, Serhat Göçer, Ümit Yıldırım, Murat Beyatlı, Cemil Emre Yurtseven, Abdullah Esmeray, Ferhat Yakup Suçeken, Ahmet Tahra, Resul Sobay, Eyüp Veli Küçük

**Affiliations:** 1 Department of Urology, Umraniye Training and Research Hospital, Istanbul 34764, Türkiye; drsametgngr@gmail.com (H.S.G.); sencersozen@gmail.com (M.S.S.); umutevci@gmail.com (M.U.E.); muratbeyatli_90@hotmail.com (M.B.); yurtsevenmd@gmail.com (C.E.Y.); esmerayabdullah@gmail.com (A.E.); ykpsckn@gmail.com (F.Y.S.); ahmettahra@gmail.com (A.T.); drresulsobay@gmail.com (R.S.); eyupveli@gmail.com (E.V.K.); 2 Urology Department, Karamanoglu Mehmetbey University Training and Research Hospital, Karaman 70100, Türkiye; drasanli07@gmail.com; 3 Department of Urology, Tekirdag City Hospital, Tekirdag 59030, Türkiye; akumcu911@hotmail.com (A.K.); serhatemir2012@gmail.com (S.G.); 4 Department of Urology, Kafkas University Training and Research Hospital, Kars 36100, Türkiye; dr.umityildirim87@gmail.com

**Keywords:** partial nephrectomy, laparoscopy, surgical margins, adherent perinephric fat, nephrometry score, tumour enucleation

## Abstract

**Background/Objectives**: Adherent perinephric fat (APN) is common in laparoscopic partial nephrectomy (LPN). It is absent from the lists of recognised positive surgical margin (PSM) predictors. We tested whether APN is associated with PSM risk and whether the effect varies by resection technique. **Methods**: We analysed 401 International Society of Urological Pathology (ISUP)-gradable renal cell carcinomas (31 PSM events; chromophobe RCC was excluded from the primary cohort because the WHO/ISUP grade is not assignable) carried out at three centres by eight surgeons from 2013 to 2024. Nested logistic models added APN, then an APN × enucleation interaction, to a guideline-based covariate set (tumour size, ISUP grade ≥ 3, cT1b stage, centre fixed effects, surgeon-documented enucleation). Firth penalisation and a 2000-replicate bootstrap were used to limit small-sample bias; they cannot add information beyond the 31 observed events. **Results**: APN was an independent PSM predictor (adjusted odds ratio [OR] 2.68, 95% confidence interval [CI] 1.21 to 5.95, *p* = 0.02). The effect was technique-conditional: APN × enucleation interaction OR 7.83 (95% CI 1.08 to 56.92, *p* = 0.04), consistent in direction across estimators but imprecise in magnitude. PSM occurred in 29.4% of APN-positive enucleation cases (odds ratio 10.21, 95% CI 2.07 to 50.30; Fisher exact *p* = 0.003), against 5.0% in enucleoresection and 10.8% in resection. **Conclusions**: In this cohort the APN-to-PSM association was technique-conditional. The penalty concentrates in enucleation; the rim-retaining strata gave estimates compatible with both no association and clinically important harm (OR 0.99 and 1.96, both intervals wide). Because the interaction rests on few margin events and the technique was not randomised, this finding is hypothesis-generating: when APN is encountered intraoperatively, retaining a parenchymal rim (enucleoresection or resection) rather than enucleation may be considered, pending prospective validation.

## 1. Introduction

Partial nephrectomy is the standard treatment for clinical T1 renal masses, and the surgeon’s task is to remove the tumour with a clean margin while preserving as much functional parenchyma as possible [1,2]. Positive surgical margin (PSM) is the oncologic endpoint of that trade-off; its association with local recurrence and progression-free survival has been variable across cohorts [3,4,5], with the strongest positive signal emerging in cohorts with extended (at least 5-year) follow-up [6]. A recent National Cancer Database analysis found that positive margins independently predicted worse overall survival and now bear on adjuvant-therapy eligibility, although this prognostic effect was stage-dependent and was not evident in stage I disease [7]. PSM occurs in two to eight percent of T1 partial nephrectomies [1].

Recognised PSM predictors in current guidelines are imperative surgical indication, adverse pathological features (pT2a, pT3a, grade III to IV), and hospital volume [1]. Single-cohort and review-level analyses have added tumour size as a paradoxical predictor [3] and surgeon experience as a modifiable factor [8]. Adherent perinephric fat (APN) does not appear in either list [1,2].

The published evidence on APN-to-PSM is null but technique-pooled. The 2021 meta-analysis pooled nine studies and 1534 patients and reported a risk ratio of 1.82 for positive margins (95% CI 0.79 to 4.18, *p* = 0.16); the estimate is null but with a wide confidence interval that does not exclude a clinically relevant effect [9]. Neither the included studies nor any other published cohort series or systematic review we identified reports the APN-to-PSM association stratified by resection technique. A technique-conditional structure, if present, would not be visible in a marginal pooled estimate. The European Association of Urology (EAU) 2026 guideline explicitly notes that evidence of the impact of resection technique on partial nephrectomy outcomes is limited by lack of standardised reporting and by the retrospective design of most available studies (level of evidence 3) [1].

We tested two pre-specified hypotheses in a contemporary multicentre LPN cohort: APN is associated with PSM independently of the guideline-based covariate set, and the association is concentrated in patients managed by surgeon-documented enucleation. The clinical aim was an intraoperative decision-frame for the operative response when APN is encountered.

## 2. Materials and Methods

### 2.1. Cohort and Design

We conducted a multicentre retrospective cohort study at three centres in Türkiye. Adults underwent elective LPN for clinical T1 solid renal masses between January 2013 and December 2024. Eight surgeons performed the procedures across the three centres. The cohort was defined by intent to treat by LPN; conversions to open or robotic approaches at the start of the case were excluded. Sex was recorded as documented in the operative notes; both sexes were included and no sex-based selection was applied.

**Figure 1:**
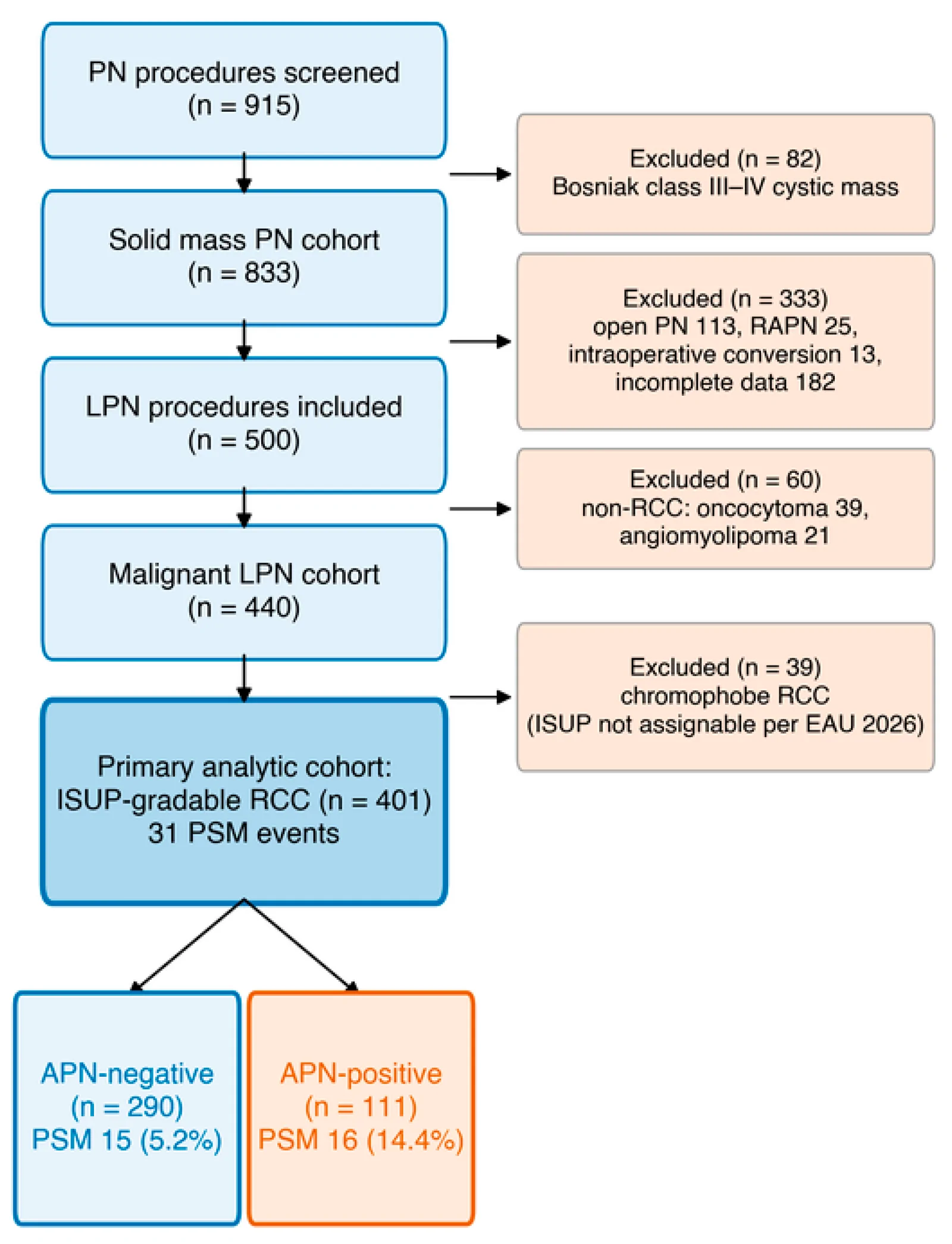
Study flow diagram (STROBE).

Of 915 patients assessed for eligibility, 82 with Bosniak class III–IV cystic masses were excluded; the study question concerns solid renal masses. Of the remaining 833 cases, 333 were excluded (open partial nephrectomy 113, robot-assisted partial nephrectomy 25, intraoperative conversion to open at the start 13, incomplete data 182), leaving 500 LPN cases. The primary analytic cohort comprised the 401 patients with renal cell carcinoma (RCC) gradable per the International Society of Urological Pathology (ISUP) system (clear cell or papillary subtype); chromophobe RCC was retained for a pre-specified sensitivity analysis only, since the WHO/ISUP grading system is not currently recommended for chromophobe tumours per the EAU 2026 guideline [1]. The full 500-patient cohort (including 39 chromophobe RCC, 39 oncocytomas, and 21 angiomyolipomas) was retained as a second pre-specified sensitivity analysis. APN-positive cases numbered 111 in the primary cohort and 139 in the full cohort. The full Strengthening the Reporting of Observational Studies in Epidemiology (STROBE) flow is in Figure 1.

### 2.2. Exposure, Outcome, and Key Derived Variable

Adherent perinephric fat was the operating surgeon’s documented intraoperative observation of inflammatory or fibrotic adhesion of perinephric fat to the renal capsule. No predefined terminology or standard descriptor was in use during the study period; surgeons recorded the finding in free text, and the two assessors applied the above definition retrospectively to that free text. APN status was extracted retrospectively from operative notes by two assessors blind to PSM status, with discrepancies resolved by consensus. Description of the perinephric plane was a routine element of the operative note at all three centres, so cases without an explicit adhesion descriptor were classified APN-negative rather than missing. The Mayo Adhesive Probability score [10] was calculated as the sum of its two components, posterior perinephric fat thickness and perinephric stranding, both scored from the preoperative cross-sectional imaging (total range 0 to 5).

Positive surgical margin was defined per the participating centres’ uniform pathology protocol as the pathologist’s binary determination of tumour cells extending beyond the pseudocapsule on the inked specimen surface (R1 status). Margin distance in millimetres, measured from the resection bed to the closest tumour edge, was recorded separately; in enucleation specimens, the retained parenchymal margin is minimal by surgical design (recorded margin distance median 0.6 mm in this cohort) (dissection along the pseudocapsule), and PSM status in this context reflects pathologist-reported tumour penetration of the pseudocapsule rather than physical distance to the ink. Effective margin (margin distance minus the local thermal artefact) was a pre-specified sensitivity outcome. Change in estimated glomerular filtration rate (eGFR) was defined as the difference between the preoperative value and the value at postoperative month 3.

Resection technique was extracted from the operative note free text as one of three categories using the surgeon’s documented description of the parenchymal dissection plane: enucleation (‘enukleasyon’ or equivalent, dissection along the tumour pseudocapsule with no intentional parenchymal rim), enucleoresection (a thin parenchymal rim retained), or resection (a wider rim, typically several millimetres). Operative-note terminology for the dissection plane was not standardised prospectively across the three centres; the three categories were defined for this study and applied uniformly by the same two assessors to the free text of every note, irrespective of centre. Both variables had been scored independently by two assessors in all 500 operative notes at the time of data collection, blind to margin status and to each other; the value analysed is the consensus of the two readings, and the agreement between them is reported here. For binary APN status, agreement was 93.8% and Cohen’s kappa was 0.84 (95% CI 0.78 to 0.89). For the three-category resection technique, agreement was 91.4%, unweighted kappa was 0.87 (95% CI 0.83 to 0.90), and linearly weighted kappa was 0.89 (95% CI 0.86 to 0.92). Within the primary analytic cohort, the corresponding values were kappa 0.84 (95% CI 0.77 to 0.90) for APN and weighted kappa 0.90 (95% CI 0.86 to 0.93) for technique. Cross-tabulations are given in Table S5.

### 2.3. Variables and Statistical Analysis

The guideline-based covariate set for adjustment of the APN-to-PSM association was pre-specified following the EAU 2026 guideline § 7.2.3.c, the American Urological Association (AUA) 2021 guideline, and supporting single-cohort and systematic-review evidence on positive surgical margin and on adherent perinephric fat [3,4,5,8,9,10,11,12]. The set comprised tumour size on imaging (continuous, centimetres) [3]; high-grade renal cell carcinoma defined as ISUP nucleolar grade ≥3 [1,4], matching Shah 2016’s high-risk pathological subgroup (Fuhrman III–IV) in current WHO/ISUP nomenclature [4]; cT1b stage versus cT1a [1]; centre fixed effects (surrogate for hospital volume) [1]; and surgeon-documented enucleation.

The RENAL (Radius, Endophytic/exophytic properties, Nearness to collecting system, Anterior/posterior, Location) [13], and Preoperative Aspects and Dimensions Used for an Anatomical (PADUA) [14] anatomical scores were tested as candidate etiologic factors and were significant on univariate logistic regression (RENAL OR 1.32 per point, 95% CI 1.07 to 1.64, *p* = 0.01; PADUA OR 1.37 per point, 95% CI 1.08 to 1.73, *p* = 0.009). Both scores were collinear with tumour size, a constituent component of either; the multivariable models retained tumour size for parsimony. cT1b stage was retained alongside continuous tumour size on a different basis: it is the stage variable specified in the guideline-based covariate set, not an alternative composite measure of the same anatomical construct. The two are nonetheless correlated (Pearson r 0.84), and Model 2 was therefore refitted omitting each in turn (Section 3.6). Sensitivity models substituting RENAL or PADUA for tumour size yielded concordant APN estimates (Table S1). Component variables of each score were not recorded individually in the database, which precluded interaction analyses with individual anatomic features.

ISUP grade ≥ 3 is determined post-operatively from the resected specimen but reflects tumour biology present at the time of surgery. In an etiologic analysis of PSM, this variable acts as a confounder of the APN-to-PSM association: high-grade tumours independently associate with peritumoral inflammation (APN positivity) and with pseudocapsule penetration (PSM positivity). ISUP grade was retained as a covariate rather than removed as a potential mediator on these a priori grounds. The exploratory within-grade analysis reported in the Results is directionally consistent with this choice, but rests on small cell counts. It is a subgroup observation. It neither establishes grade as a confounder nor settles whether grade lies on the pathway from APN to PSM. The classification rests on the assumed causal structure (Figure S2). In any case, postoperative grade is unknown at the moment the surgeon selects the resection plane, so it cannot enter an intraoperative decision rule. Sarcomatoid and rhabdoid differentiation are equivalent to ISUP grade 4 by the WHO/ISUP convention [1] and are not modelled separately. The full directed acyclic graph is presented in Figure S2.

The primary descriptive estimate was the univariate APN-to-PSM odds ratio (OR), with Fisher exact *p* values. The covariate set, nested logistic models, APN × enucleation interaction test, and likelihood-ratio comparisons were specified prior to data analysis; the analysis plan was not publicly registered. Three pre-specified nested logistic regression models were fitted in sequence. Model 1 contained the guideline-based covariate set without APN, Model 2 added APN to the guideline-based baseline, and Model 3 added the APN-by-enucleation interaction term. Pre-specified likelihood-ratio tests compared nested models (Model 2 vs. Model 1 for APN main effect, Model 3 vs. Model 2 for interaction). The events-per-variable ratio was 5.2 for Model 1, 4.4 for Model 2, and 3.9 for Model 3; we therefore also fitted Firth penalised logistic regression [15] and a 2000-replicate non-parametric percentile bootstrap. These procedures address small-sample and separation-induced bias only; they do not correct residual confounding, exposure misclassification, or the non-randomised allocation of resection technique. With only 31 PSM events and 3.9 events per variable, Model 3 is statistically unstable and at risk of overfitting. The interaction is carried by two sparse cells: 10 of 34 APN-positive against 2 of 51 APN-negative enucleation cases. Its estimates should be read accordingly. Robustness analyses comprised leave-one-surgeon-out, centre-stratified estimates, sensitivity analysis on the full 500-patient cohort (including benign histology), addition of surgeon experience (cumulative case count, range 60 to 180 per surgeon) to Model 3 as Model 4 to test for residual confounding by operator learning curve, and an E-value for the magnitude of unmeasured confounding that would explain the association [16]. To account for possible clustering of outcomes within surgeons, Model 3 was also estimated with cluster-robust standard errors grouped by surgeon. Because tumour size and cT1b stage are correlated, Model 2 was refitted omitting each in turn to confirm the APN estimate did not depend on their joint inclusion. Two-sided *p* < 0.05 was the threshold of statistical significance throughout. Analyses were conducted in Python 3.14 with pandas 2.2.3 and statsmodels 0.14; Firth penalised logistic regression was implemented by iteratively reweighted modified Newton–Raphson on the Jeffreys-prior-penalised log-likelihood. Manuscript drafting and Python code preparation were supported by a large language model (Claude Sonnet-5, Anthropic, San Francisco, CA, USA); all analytic decisions, statistical interpretations, and final manuscript text are the authors’ own.

## 3. Results

### 3.1. Cohort

The primary analytic cohort comprised 401 ISUP-gradable LPN cases (clear cell 340 and papillary 61) operated by eight surgeons at three centres; APN was operator-documented in 27.7% (111/401) of cases (APN-positive *n* = 111, APN-negative *n* = 290). Median age was 61 years, 63% male, median body mass index 28.6 kg/m^2^. Median tumour size on imaging was 3.2 cm and 30% were cT1b. Median RENAL was 6 and PADUA was 7. High-grade RCC (ISUP grade ≥ 3) was observed in 24.2% (97/401) of cases (clear cell 25.0%, papillary 19.7%). Median follow-up was 44.1 months (interquartile range [IQR] 26.5 to 64.2). The APN-positive and APN-negative groups were balanced across baseline demographic, comorbidity, tumour, and complexity-score variables (Table 1). The two groups differed primarily in PSM frequency (14.4% vs. 5.2%, Fisher exact *p* = 0.003) and in the prevalence of high-grade RCC (37.8% in APN+ vs. 19.0% in APN−, *p* < 0.001).

### 3.2. APN-to-PSM Association

APN-positive cases carried a substantially higher PSM risk than APN-negative cases. PSM occurred in 16 of 111 APN-positive cases (14.4%) and 15 of 290 APN-negative cases (5.2%; Fisher exact *p* = 0.003, univariate OR 3.09, 95% confidence interval (CI) 1.47 to 6.48). The E-value for the univariate odds ratio was 5.63, indicating that an unmeasured confounder would need to be associated with both APN and PSM by odds ratios of approximately 5.6 to explain the observed association.

### 3.3. Guideline-Based Adjusted Estimates (Models 1 and 2)

In the guideline-based Model 1, the dominant PSM predictor was high-grade RCC (adjusted OR 2.87, 95% CI 1.31 to 6.25, *p* = 0.008), followed by surgeon-documented enucleation (adjusted OR 2.39, 95% CI 1.07 to 5.33, *p* = 0.03). Tumour size, cT1b stage, and centre fixed effects were not statistically significant in this model. Adding APN to the guideline-based covariate set (Model 2) produced an APN adjusted OR of 2.68 (95% CI 1.21 to 5.95, *p* = 0.02). The association persisted above the recognised guideline-based set. The likelihood-ratio test (Model 2 vs. Model 1) yielded chi-square 5.72, degrees of freedom (df) = 1, *p* = 0.02. Adjusted estimates from Models 1, 2, and 3 are summarised in Table 2.

### 3.4. APN × Enucleation Interaction (Model 3)

Model 3 added the APN-by-enucleation interaction term. The interaction reached the conventional threshold but was imprecise (adjusted interaction OR 7.83, 95% CI 1.08 to 56.92, *p* = 0.04; likelihood-ratio test Model 3 vs. Model 2 chi-square 4.79, df = 1, *p* = 0.03). The interval spans a more than fiftyfold range. The point estimate of 7.83 should not be read as an established effect size. The Firth estimate was OR 5.97 (95% CI 0.96 to 37.10) (Table S2). The interaction is also fragile to single-event reclassification. Reclassifying any one of the 10 positive margins in the APN-positive enucleation cell as margin-negative moved the interaction *p* value above 0.05 in all 10 permutations (OR 5.95 to 7.18, *p* = 0.053 to 0.078). Reclassifying any one APN-negative enucleation case as a positive margin did the same in all 49 permutations (OR 4.65 to 5.35, *p* = 0.069 to 0.096). The point estimate remained above 1 throughout. The direction is stable; the significance is not. In Model 3, neither APN nor enucleation carried an independently significant main effect when the other was held at its reference level (APN within non-enucleation: OR 1.44, 95% CI 0.52 to 4.03, *p* = 0.48; enucleation within APN-negative: OR 0.63, 95% CI 0.13 to 2.99, *p* = 0.56): a pattern characteristic of a synergistic interaction in which neither factor carries the elevated risk alone. Model 2 therefore reports the cohort average and Model 3 the technique-specific structure underneath it. Model discrimination improved across the nested sequence, with C-statistics of 0.728, 0.771, and 0.784 for Models 1, 2, and 3 and Akaike information criteria of 210.4, 206.7, and 203.9; the Bayesian information criterion did not improve (238.4, 238.6, and 239.8), so the two information criteria disagree at this sample size (Table 2). These C-statistics are apparent, in-sample values. At 31 events they are optimistic. The improvement from Model 2 to Model 3 is a gain in fit within this cohort, not demonstrated out-of-sample discrimination. The Firth penalisation, which shrank the interaction odds ratio from 7.83 to 5.97, is a direct indication that the unpenalised estimate is inflated by sparse-data bias.

### 3.5. APN × Technique Stratum-Specific Estimates

**Figure 2:**
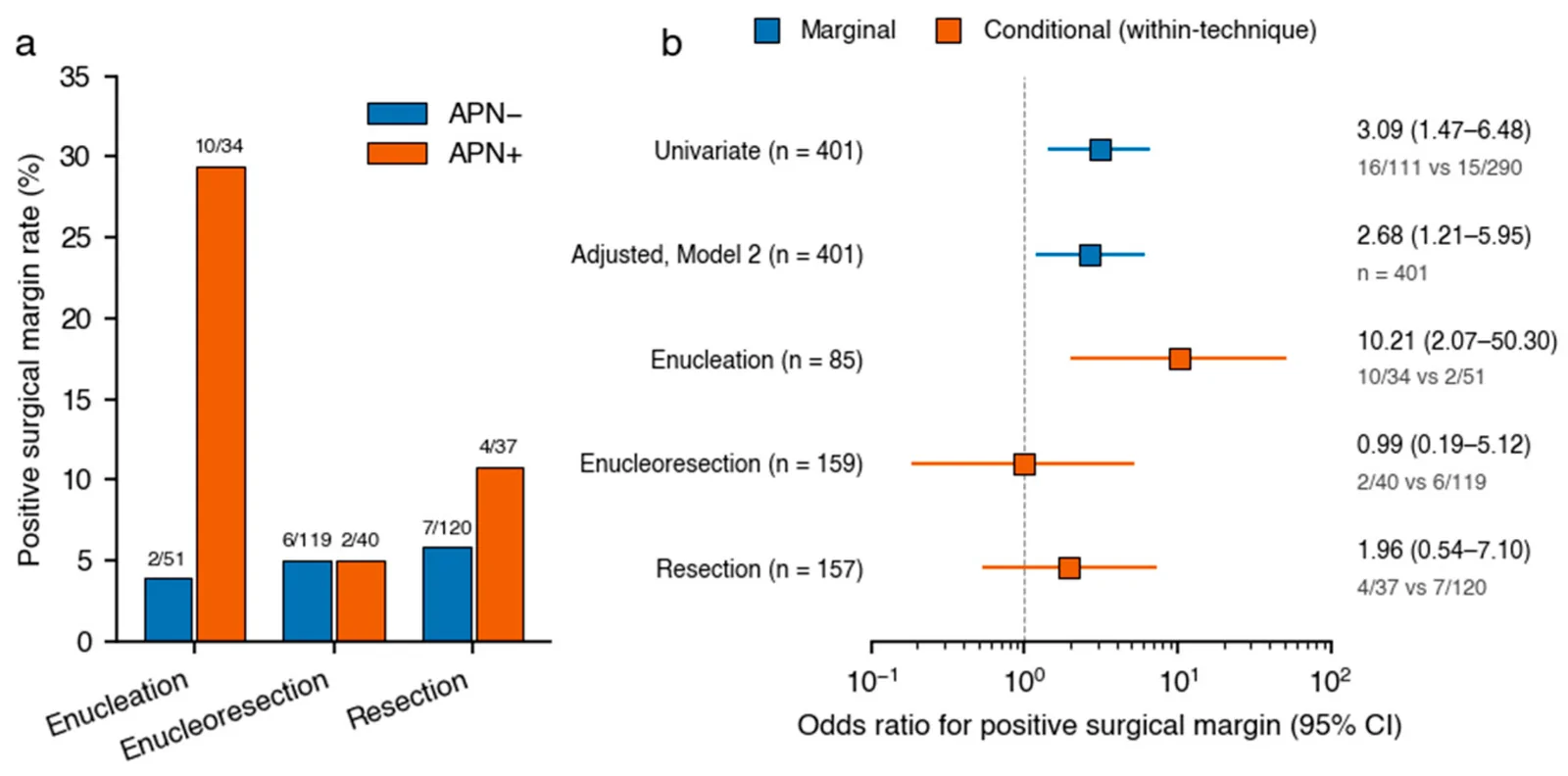
Two-panel display of the APN-to-PSM association. (**a**) Positive surgical margin (PSM) rate by adherent perinephric fat (APN) status and surgeon-documented resection technique (enucleation, enucleoresection, resection); numbers above bars indicate positive surgical margin events over subgroup n. (**b**) Forest plot of APN odds ratios (log scale) for PSM across five estimators, grouped into a marginal panel (univariate, primary cohort; Model 2 adjusted, primary cohort) and a conditional panel (within-enucleation, within-enucleoresection, within-resection). The dashed vertical line marks OR = 1 (no effect); subgroup sample sizes and, where applicable, the APN-positive versus APN-negative event counts are printed beside each estimate. Numerical estimates are reported in the Results and in Table 2.

Stratum-specific odds ratios were consistent with the interaction term. In the enucleation stratum, PSM occurred in 10 of 34 APN-positive cases (29.4%) and 2 of 51 APN-negative cases (3.9%; OR 10.21, 95% CI 2.07 to 50.30, *p* = 0.003). In the enucleoresection stratum, PSM occurred in 2 of 40 APN-positive cases (5.0%) and 6 of 119 APN-negative cases (5.0%; OR 0.99, 95% CI 0.19 to 5.12, *p* > 0.99). In the resection stratum, PSM occurred in 4 of 37 APN-positive cases (10.8%) and 7 of 120 APN-negative cases (5.8%; OR 1.96, 95% CI 0.54 to 7.10, *p* = 0.29). The technique-stratified pattern is summarised in Figure 2 (panel a, stratum PSM rates; panel b, marginal and conditional odds ratios).

In a subgroup analysis restricted to rim-retaining techniques (*n* = 316), the univariate APN-PSM odds ratio was 1.47 (95% CI 0.54 to 4.01, Fisher exact *p* = 0.42); no APN-associated excess was detected outside the enucleation stratum, although this interval is wide and does not exclude a modest effect.

An additional descriptive pattern emerged within the primary cohort. APN’s incremental PSM penalty was concentrated in low-grade tumours: PSM occurred in 6 of 235 APN-negative ISUP 1–2 cases (2.6%) and 9 of 69 APN-positive ISUP 1–2 cases (13.0%), whereas the APN penalty narrowed in ISUP ≥3 tumours (16.4% APN-negative vs. 16.7% APN-positive). The APN × ISUP-grade multiplicative interaction term was inverse (OR 0.18, 95% CI 0.04 to 0.82, *p* = 0.03; Figure S1), indicating that the APN-associated PSM elevation was attenuated in tumours whose underlying grade already carries an elevated PSM risk. This APN × ISUP-grade pattern is exploratory and hypothesis-generating; given the small cell counts and the multiple comparisons performed, it should be interpreted with caution.

### 3.6. Robustness and Sensitivity

Robustness analyses were consistent with the primary estimates. Leave-one-surgeon-out univariate odds ratios for APN ranged from 2.41 to 4.07 (median 2.96). Centre-stratified analysis yielded a centre-adjusted APN OR of 3.02 (95% CI 1.44 to 6.36, *p* = 0.004). Sensitivity analysis on the full 500-patient cohort yielded a univariate APN OR of 2.87 (95% CI 1.49 to 5.51), with an adjusted OR of 2.46 (95% CI 1.22 to 4.97). Sensitivity analysis on the all-malignant cohort (*n* = 440, primary cohort plus 39 chromophobe RCC; 35 PSM events) yielded a univariate APN OR of 3.10 (95% CI 1.54 to 6.25, *p* = 0.002). Non-parametric bootstrap (2000 replicates) yielded a univariate APN median OR of 3.12 (95% CI 1.37 to 7.08). The pre-specified effective-margin outcome was narrower in APN-positive cases (median 0.9 versus 1.5 mm, Mann–Whitney *p* = 0.03). Within the enucleation stratum it did not differ between APN groups, and both medians were below zero (−0.6 versus −0.8 mm, *p* > 0.99). Surgeon experience (cumulative case count, range 60 to 180 partial nephrectomies per surgeon) was added to Model 3 as a sensitivity covariate (full output in Table S3): the APN main-effect OR changed from 1.44 to 1.46, the APN × enucleation interaction OR changed from 7.83 to 7.79, and the likelihood-ratio test for the experience contribution was not significant (chi-square 0.07, df = 1, *p* = 0.80). Stratum-specific PSM rates in APN-positive enucleation cases were 21.4%, 20.0%, and 50.0% across low, mid, and high experience tertiles. The Mayo Adhesive Probability score discriminated APN status well in this cohort (area under the curve [AUC] 0.84, 95% CI 0.80 to 0.88); the proportion of APN-positive cases rose across score categories from 3.2% at a score of 0 to 80.0% at a score of 5, comparable in direction to earlier validation studies [17,18]. Smaller tumours showed a comparable APN-associated PSM penalty across size cutoffs (10.7 percentage points at ≤3 cm vs. 8.2 at >3 cm; 8.3 percentage points at ≤4 cm vs. 12.4 at >4 cm). The descriptive polar-location pattern showed a gradient of APN penalty (14.7 percentage points at the upper pole, 11.5 at the mid pole, 2.2 at the lower pole). Long-term oncologic outcomes were sparse over the median 44.1-month follow-up: 17 local recurrences, 14 distant metastases, and 10 deaths. Crude rates in PSM-positive versus PSM-negative cases were 6.5% versus 4.1% for local recurrence and 6.5% versus 3.2% for distant metastasis. With cluster-robust standard errors grouped by surgeon, the APN × enucleation interaction odds ratio was unchanged at 7.83 (95% CI 1.38 to 44.45, *p* = 0.02). Eight surgeons are too few clusters for that interval to be reliable. Its narrowness reflects the anti-conservative behaviour of cluster-robust standard errors at this number, not added precision. The APN estimate in Model 2 was unchanged when tumour size or cT1b stage was omitted (adjusted OR 2.65 to 2.68). APN-positive and APN-negative cases did not differ in warm ischaemia time, estimated blood loss, operative time, conversion, or intraoperative complications (all *p* > 0.10). These were not equivalence tests, and an intraoperative-difficulty gradient is therefore not excluded. Within the 111 APN-positive cases, the change in eGFR from baseline to postoperative month 3 did not differ between enucleation (*n* = 34; median −7.2, IQR −12.5 to −1.4 mL/min/1.73 m^2^) and rim-retaining techniques (*n* = 77; median −5.5, IQR −8.9 to −1.6; Mann–Whitney *p* = 0.60); no values were missing. Clavien–Dindo grade ≥3 complications were no more frequent when a rim was retained (4 of 77, 5.2%, versus 3 of 34, 8.8%; Fisher exact *p* = 0.67). The analyses by tumour-size cutoff, polar location, and experience tertile were exploratory and unadjusted for multiple comparisons.

## 4. Discussion

In this cohort, the APN-to-PSM association was technique-conditional. It rests on 31 margin events, 12 of them in the enucleation stratum. APN carried an adjusted risk above the guideline-based covariate set (OR 2.68, 95% CI 1.21 to 5.95), but that cohort-average figure is misleading in the operating room. The elevated risk in APN-positive cases was observed only in the enucleation stratum (within-enucleation OR 10.21, 95% CI 2.07 to 50.30). It was not detected when a parenchymal rim was retained (within-enucleoresection OR 0.99; within-resection OR 1.96), although these rim-retaining strata are small and their confidence intervals wide. The direction of the pattern was consistent across three estimators (standard maximum-likelihood, Firth penalised, and bootstrap) and across leave-one-surgeon-out, centre-stratified, and full-cohort sensitivity analyses, although its magnitude was not. The interaction is estimated with low precision. Model 3 carries 31 margin events and 3.9 events per variable. The point estimate moved from 7.83 under maximum likelihood to 5.97 under Firth and 7.20 as the bootstrap median, and the confidence interval spans 1.08 to 56.92. Three features nonetheless support the qualitative conclusion. The covariate set and the interaction term were specified in advance rather than selected from the data. The likelihood-ratio test for the interaction (chi-square 4.79, df = 1, *p* = 0.03) does not depend on the width of the Wald interval. The stratum-specific contrast within enucleation (10 of 34 versus 2 of 51; odds ratio 10.21, 95% CI 2.07 to 50.30; Fisher exact *p* = 0.003) is model-free. The Firth Wald *p* value for the interaction sits at the boundary (*p* = 0.06), so the claim we make is that the interaction is directionally robust, not that its magnitude is established. The E-value is the smallest association an unmeasured confounder would need to have with both adherent perinephric fat and positive surgical margin, on the odds-ratio scale, to explain the observed association away: 4.80 for the adjusted estimate and 5.63 for the unadjusted one.

APN was not listed among recognised PSM predictors in either the EAU 2026 guideline or the AUA 2021 guideline. The 2021 meta-analysis pooled nine studies and 1534 patients and reported a null APN-to-PSM association (RR 1.82, 95% CI 0.79 to 4.18) [9], but the analysis was technique-pooled. Single-cohort series reporting null APN-to-PSM associations [19] did not record the resection plane, and neither did the studies pooled in the meta-analysis [9]; none could therefore test interaction with surgeon-documented enucleation. Our cohort, with techniques distributed across the three categories (enucleation 21%, enucleoresection 40%, resection 39%), provided sufficient enucleation cases to populate the interaction term that pooled estimates obscure. The discrepancy between our marginal APN OR (3.09 univariate) and the technique-pooled meta-analytic estimate (RR 1.82, 95% CI 0.79 to 4.18 [9]) is consistent with this composition-dependent interpretation: our cohort’s enucleation prevalence of 21% was sufficient to populate the interaction term and reveal the conditional structure that the meta-analytic pool obscured. A direct quantitative reconciliation supports this interpretation: restricting our analysis to rim-retaining techniques (subgroup cohort, *n* = 316) reproduces the meta-analytic null almost exactly (APN OR 1.47, 95% CI 0.54 to 4.01, *p* = 0.42), whereas adding the enucleation stratum back recovers the conditional signal. Khene’s pooled estimate is therefore not in conflict with our finding. A marginal estimate averages over whatever technique distribution the pooled cohorts had, and that distribution was not recorded.

The EAU 2026 guideline explicitly notes that the evidence on the impact of resection technique on PN outcomes is limited by lack of standardised reporting and by the retrospective design of most available studies (level of evidence 3). Our analysis addresses one specific aspect of this gap: the interaction between an intraoperative finding (APN) and a surgeon-documented technique.

A surgical-mechanism interpretation is consistent with the stratified pattern. Enucleation is defined by dissection along the tumour pseudocapsule with no intentional parenchymal rim [20,21,22,23]. When the peri-renal plane is anatomically clean, the pseudocapsule is reliably identifiable and the dissection achieves a defined, if minimal, oncologic margin. When perinephric fat is inflamed and adherent, the peri-renal plane is altered and the pseudocapsule’s intraoperative identifiability is compromised. Enucleoresection and resection, by retaining a rim of normal parenchyma, preserve a buffer that absorbs this uncertainty; enucleation does not. The mechanism predicts no APN penalty in the rim-retaining techniques and a concentrated penalty in enucleation: consistent with the observed pattern, although the rim-retaining strata are too small and their intervals too wide to exclude an APN effect (stratum-specific APN OR 10.21 within enucleation, 0.99 within enucleoresection, 1.96 within resection). A similar pattern was anticipated by Yossepowitch and colleagues [3], who observed positive margins more often in smaller tumours and hypothesised surgeon reluctance to incise a generous parenchymal rim in small, exophytic lesions. Enucleation operationalises that reluctance—it is the explicit decision to forego the rim. In APN-positive cases managed this way, margin failure was more frequently observed.

The nature of the exposure warrants comment. The Mayo Adhesive Probability score, a radiological proxy for perinephric fat, discriminated documented APN well (AUC 0.84), and APN-positive cases did not differ from APN-negative cases in warm ischaemia time, blood loss, operative time, or complications. Documented APN therefore tracks the radiological fat burden. Those comparisons were not equivalence tests and do not exclude a difficulty gradient. The label also reflects the surgeon’s specific observation of inflammatory or fibrotic adhesion that may compromise pseudocapsule identifiability. The operating surgeon records the label in the same note that records the dissection plane. Part of what it captures may therefore be difficulty met during that dissection rather than a tissue property present before it. The exposure cannot be assumed independent of the technique used, and this ambiguity limits the causal reading of the interaction. This subjective ascertainment is a limitation, and exposure misclassification cannot be excluded. The published score is itself reproduced only fairly between readers, and its stranding component is graded subjectively [18]. The validation cohorts [17,18] were open partial nephrectomy series with a different case mix and a radiologist-scored exposure. In our cohort, APN was recorded without a standardised intraoperative definition, so documentation may have varied between the eight surgeons. The two measures agree substantially, which supports the surgeon-documented label as a marker of perinephric fat rather than of operative difficulty.

The conditional structure suggests a hypothesis for intraoperative decision-making rather than a validated rule. When APN is encountered, the resection plane is one parameter that may warrant reconsideration: in this cohort, retaining a parenchymal rim (enucleoresection or resection) was associated with a lower APN-related PSM risk than enucleation. A surgeon planning enucleation for a small or low-complexity tumour might therefore weigh an enucleoresection or resection rim once the perinephric plane is judged inflamed or adherent. The descriptive magnitude was 29.4% PSM in APN-positive enucleation, against 5.0% in enucleoresection and 10.8% in resection. Because technique was not randomised and the enucleation stratum contributed only 12 margin events, this inference is hypothesis-generating and requires prospective validation before it can guide practice.

A preference for a wider rim must weigh the functional cost that enucleation is designed to avoid. That cost was not apparent here: within APN-positive cases, the change in eGFR did not differ between enucleation and rim-retaining techniques, and higher-grade complications were no more frequent when a rim was retained. Post-partial-nephrectomy renal function is multifactorial. Preoperative eGFR, sex, ischaemia technique, and early postoperative function carried the strongest support as predictors of one-year decline in a multimodel comparison [24]; resection volume was not among the variables evaluated. The small functional difference we observed sits within that picture. These functional data are observational and hypothesis-generating, but they indicate that retaining a rim when APN is encountered need not carry a large functional penalty.

Two methodological caveats qualify the technique finding. First, technique allocation in this cohort was non-random; surgeons chose the plane based on intraoperative judgement, which may correlate with unobserved features that also bear on PSM risk (confounding by indication). Tumour size and the RENAL and PADUA totals capture only part of that judgement. Hilar tumour location, a completely endophytic growth pattern, and proximity to the collecting system may each push a surgeon toward or away from enucleation while independently raising margin risk. Only total nephrometry scores were recorded, so these features could not be adjusted for. Residual confounding by indication cannot be excluded. Both the APN label and the technique label come from the same operating surgeon and the same operative note, so exposure and treatment are not ascertained independently of each other. The interaction may therefore partly reflect which APN-positive tumours a surgeon elected to enucleate rather than an effect of the plane itself. Second, surgeon-documented enucleation and pathologist-reported margin distance are aligned by surgical convention: enucleation implies dissection along the pseudocapsule, which yields the narrowest margin by design. The guideline-based Model 1 result that enucleation carries an OR of 2.39 for PSM independent of APN reflects this baseline narrowness. What the APN term contributes in Models 2 and 3 is a further increment above this baseline: stratum-specific PSM rises from 3.9% to 29.4% within enucleation, while remaining flat within enucleoresection (5.0%) and modestly elevated within resection (5.8% to 10.8%): a joint effect not explained by technique or APN alone. The cohort’s eight surgeons were uniformly above the literature-cited 30-case experience threshold for partial nephrectomy [8]; adding surgeon experience as a covariate to Model 3 did not materially change the APN main effect or the APN × enucleation interaction (Model 4 sensitivity), although an objective surgeon-level metric capturing training quality, fellowship subspecialisation, and institutional learning curves would strengthen replication. Eight surgeons contributed cases over twelve years. They may have differed in how reliably they recognised and documented APN, in where they placed the boundary between enucleation and enucleoresection, in their preferred technique, and in their individual PSM rates (Table S3). Practice may also have changed between 2013 and 2024. The leave-one-surgeon-out analysis tests whether any single operator drives the pooled estimate; it does not remove this between-surgeon heterogeneity, and neither centre fixed effects nor cumulative case volume substitutes for operator identity.

PSM is a surrogate endpoint, not a patient outcome. It was retained as the primary endpoint of this analysis, but its long-term oncologic significance was not formally tested in this cohort because of a relatively low event count (17 local recurrences, 14 distant metastases, 10 deaths) and a median follow-up of 44.1 months (IQR 26.5 to 64.2) that, while comparable to other PSM cohorts (33 months in Shah 2016 [4]; mean 37 months in Bensalah 2010 [5]; range 24 to 61 months across the eight studies pooled in the Henderickx 2022 systematic review [12]), falls substantially short of the 96-month median in Tellini 2019 [6], the cohort that produced the strongest recent positive PSM-to-progression-free survival (PFS) signal (adjusted odds ratio 3.13, *p* = 0.01). Descriptive crude rates in our cohort (local recurrence 6.5% in PSM-positive versus 4.1% in PSM-negative; distant metastasis 6.5% versus 3.2%) suggest a direction of effect consistent with Tellini 2019 but are too sparse to support formal inference. The EAU 2026 guideline recommends intensified follow-up for patients with PSM after nephron-sparing surgery (weak recommendation) [1]; our analysis is consistent with that recommendation but does not have the follow-up duration required to independently confirm or refute the long-term PSM-to-PFS association.

The mechanism we propose concerns the tissue and the resection plane rather than the access platform. A robotic series bears on this. In the Surface-Intermediate-Base Margin Score Consortium, margin risk was not ordered along the plane: the intermediate enucleoresection technique carried the highest rate (11.4%), above both enucleation (4.5%) and resection (3.6%), and only the enucleoresection-versus-enucleation contrast was independent on multivariable analysis [25]. Whether the APN-conditional pattern we observed under laparoscopy holds under robotic assistance is therefore an open question that prospective robotic cohorts should address.

We report this multicentre retrospective cohort study according to the STROBE recommendations for observational research [26]. Several threats to inference remain. They are observer bias in APN ascertainment, instability and possible overfitting of the interaction model (3.9 events per variable), sparse events in the high-complexity stratum (one patient under the Kutikov [13] and Ficarra [14] original strata), single-region generalisability, absence of RENAL and PADUA component data (only total scores were recorded, which precluded interaction analyses with individual anatomic features), and the observational technique-allocation noted above. APN was extracted blind to PSM status from operative notes, but no prospective checklist or video validation was used; an objective intraoperative APN scoring system would strengthen replication [27]. Bosniak class III–IV cystic masses were excluded a priori; their margin dynamics differ from solid masses and require separate evaluation. The cohort is restricted to three Turkish centres in 2013–2024 and to laparoscopic technique; extension to robotic partial nephrectomy and to non-Turkish settings is open. All eight surgeons exceeded the 30-case experience threshold cited in Carbonara 2022 [8]; whether the technique-conditional pattern generalises to lower-volume community settings (where partial-nephrectomy case volume per surgeon is below this threshold) is not addressed by this cohort. The 182 cases excluded for incomplete data (22% of the eligible solid-mass cohort) could not be formally compared with included cases. Their records were not retained in the analytic dataset and could not be recovered from the hospital records, so neither the pattern of missingness nor the direction of any selection bias can be established. This is an important limitation: a complete-case cohort excluding more than one in five eligible patients may differ from the source population in ways this analysis cannot detect, and the estimates should be read with that in mind. Several secondary analyses were exploratory: the APN × grade interaction, tumour-size cutoffs, polar location, and experience tertiles. They were unadjusted for multiple comparisons and are reported as hypothesis-generating rather than confirmatory. The number of tests performed inflates the risk that one or more of these findings is a false positive.

## 5. Conclusions

Adherent perinephric fat was associated with positive surgical margin in a technique-conditional pattern. The penalty concentrates in enucleation; the rim-retaining strata gave estimates compatible with both no association and clinically important harm, and those strata are small. The direction of this technique-conditional pattern is robust across estimators; its magnitude is not precisely estimated. What looks like a cohort-wide APN effect is the weighted average of a technique-specific signal carried by a single resection plane. These findings are hypothesis-generating: they suggest that, when APN is encountered intraoperatively, retaining a parenchymal rim—rather than changing the approach or the ischaemia strategy—merits prospective study. External, prospective validation is required before an intraoperative APN finding should modify resection strategy; whether the excess margin risk we describe translates into recurrence or survival cannot be answered at 44.1 months of median follow-up.

## Figures and Tables

**Table 1:** Baseline characteristics by adherent perinephric fat status (primary cohort: ISUP-gradable malignant RCC, *n* = 401).

Variable	APN− (*n* = 290)	APN+ (*n* = 111)	Effect (95% CI)	*p* Value
Age, years, median (IQR)	60.5 (53.2–69.0)	62.0 (55.0–70.0)	Δ 1.0 (−1.0 to 4.0)	0.31
Male sex, *n* (%)	180 (62.1)	72 (64.9)	OR 1.13 (0.71–1.78)	0.65
Body mass index, kg/m^2^, median (IQR)	28.5 (24.6–31.9)	28.9 (25.4–32.2)	Δ 0.5 (−0.6 to 1.7)	0.36
Charlson Comorbidity Index, median (IQR)	2 (1–3)	2 (1–3)	Δ 0 (−1 to 0)	0.25
Tumour size on imaging, cm, median (IQR)	3.2 (2.3–4.7)	3.0 (1.8–4.6)	Δ −0.2 (−0.6 to 0.1)	0.22
cT1b stage, *n* (%)	88 (30.3)	31 (27.9)	OR 0.89 (0.55–1.44)	0.71
RENAL nephrometry score, median (IQR)	6 (5–7)	6 (5–7)	Δ 0 (−1 to 0)	0.22
ISUP grade ≥ 3, *n* (%)	55 (19.0)	42 (37.8)	OR 2.60 (1.60–4.22)	<0.001

Continuous variables: median (interquartile range), Mann–Whitney U test. Categorical variables: n (%), Fisher exact or chi-square test. Effect column: continuous variables—Hodges–Lehmann median difference (Δ, APN+ minus APN−) with percentile bootstrap 95% CI (2000 iterations); categorical variables—odds ratio (OR, APN+ vs. APN−) with Wald 95% CI. Additional baseline characteristics not displayed here were comparable between APN-positive and APN-negative groups and are presented in Table S4. Positive surgical margin rates are reported in the Results and in Figure 2a, and the multivariable adjustment in Table S1. APN, adherent perinephric fat; ISUP, International Society of Urological Pathology.

**Table 2:** Pre-specified guideline-based multivariable logistic models decomposing the APN-to-PSM association into marginal (cohort-average) and conditional (technique-specific) components in the primary cohort (ISUP-gradable malignant RCC, *n* = 401, 31 PSM events).

Predictor	Model 1 Adj OR (95% CI); *p* Value	Model 2 (+APN) Adj OR (95% CI); *p* Value	Model 3 (+Interaction) Adj OR (95% CI); *p* Value
Guideline-based covariates			
High-grade RCC (ISUP grade ≥ 3)	2.87 (1.31–6.25); *p* = 0.008	2.50 (1.15–5.47); *p* = 0.02	2.19 (0.98–4.88); *p* = 0.06
Surgeon-documented enucleation	2.39 (1.07–5.33); *p* = 0.03	2.02 (0.89–4.62); *p* = 0.09	0.63 (0.13–2.99); *p* = 0.56 ^a^
Exposure			
Adherent perinephric fat (APN)	—	2.68 (1.21–5.95); *p* = 0.02	1.44 (0.52–4.03); *p* = 0.48 ^a^
APN × enucleation interaction	—	—	7.83 (1.08–56.92); *p* = 0.04
Model fit			
Log-likelihood	−98.20	−95.34	−92.95
LR test vs. previous model	—	χ^2^ = 5.72, df = 1, *p* = 0.02	χ^2^ = 4.79, df = 1, *p* = 0.03
AIC	210.4	206.7	203.9
BIC	238.4	238.6	239.8
C-statistic (AUC)	0.728	0.771	0.784

Notes. Multivariable logistic regression (maximum likelihood). Adjusted odds ratios (OR) with 95% Wald confidence intervals. Model 1 contains the guideline-based covariate set without APN, following the European Association of Urology 2026 guideline § 7.2.3.c [1], the American Urological Association 2021 guideline [2], and supporting single-cohort and systematic-review evidence on positive surgical margin and on adherent perinephric fat [3,4,5,8,9,10,11,12]. Model 2 adds APN as the exposure. Model 3 adds the APN × enucleation interaction term. Models also adjusted for tumour size, cT1b stage, and centre fixed effects (nested adjustment in Table S1; full Firth penalised output of all covariates in Table S2). Likelihood-ratio (LR) tests compare nested models. All models were also fitted by Firth penalised logistic regression and a 2000-replicate non-parametric percentile bootstrap (full output in Table S2). ^a^ In Model 3, the APN and enucleation main-effect coefficients are the effects of each factor when the other is at its reference level (non-enucleation; APN-negative); the interaction term gives the multiplicative excess risk when both are jointly present; — = not estimated. APN, adherent perinephric fat; PSM, positive surgical margin; df, degrees of freedom.

## Data Availability

The data presented in this study are available on request from the corresponding author. The data are not publicly available owing to privacy and ethical restrictions.

## Supplementary Materials

The following supporting information can be downloaded at: https://www.mdpi.com/article/10.3390/jcm15176884/s1, Table S1: nested adjustment, technique-stratified, centre-stratified, and sensitivity analyses; Table S2: Firth penalised logistic regression models (Models 1–3) and 2000-replicate bootstrap distribution; Table S3: surgeon-experience confounder test (Model 4); Table S4: full baseline characteristics; Table S5: inter-rater agreement for APN status and resection technique; Figure S1: positive surgical margin rate by APN status stratified by ISUP nucleolar grade; Figure S2: hypothesised directed acyclic graph of the APN-to-PSM relationship.

## Abbreviations

The following abbreviations are used in this manuscript:

AIC	Akaike information criterion
APN	Adherent perinephric fat
AUA	American Urological Association
AUC	Area under the curve
BIC	Bayesian information criterion
CI	Confidence interval
df	Degrees of freedom
EAU	European Association of Urology
eGFR	Estimated glomerular filtration rate
IQR	Interquartile range
ISUP	International Society of Urological Pathology
LPN	Laparoscopic partial nephrectomy
LR test	Likelihood-ratio test
OR	Odds ratio
PADUA	Preoperative Aspects and Dimensions Used for an Anatomical classification
PFS	Progression-free survival
PN	Partial nephrectomy
PSM	Positive surgical margin
RCC	Renal cell carcinoma
RENAL	Radius, Endophytic/exophytic properties, Nearness to collecting system, Anterior/posterior, Location relative to polar lines (nephrometry score)
RR	Risk ratio
STROBE	Strengthening the Reporting of Observational Studies in Epidemiology.
WHO	World Health Organization
