# Supplementary material for: Adherent Perinephric Fat and Positive Surgical Margin in Laparoscopic Partial Nephrectomy: A Technique-Conditional Association Concentrated in Enucleation
Source: J Clin Med. 2026 Sep 5;15(17):6884. doi: 10.3390/jcm15176884 (PMC13566147; doi:10.3390/jcm15176884)
Supplement: Supplementary file 1 [file jcm-15-06884-s001.zip › jcm-4431452-supplementary.pdf]

Supplementary Materials

Adherent perinephric fat and positive surgical margin in laparoscopic partial nephrectomy: a technique-conditional association concentrated in enucleation

Table S1. Nested multivariable adjustment, technique-stratified, centre-stratified, and sensitivity analyses for the APN-to-PSM association (primary cohort: ISUP-gradable malignant RCC, n = 401, 31 PSM events)

Analysis	Adjustment/stratum	APN OR (95% CI)	p value
Nested multivariable adjustment (logistic regression, primary cohort)			
Univariate	Unadjusted (Fisher exact p)	3.09 (1.47–6.48)	0.003
Model A	APN univariate (logistic)	3.09 (1.47–6.48)	0.003
Model B	+ tumour size (continuous)	3.31 (1.56–7.03)	0.002
Model C	+ size + high_grade (ISUP ≥3)	2.95 (1.36–6.42)	0.006
Model D	+ size + high_grade + cT1b	3.01 (1.38–6.55)	0.006
Model E	+ size + high_grade + cT1b + centre	2.96 (1.36–6.45)	0.006
Model F	+ size + high_grade + cT1b + centre + enucleation	2.68 (1.21–5.95)	0.02
APN OR stratified by surgeon-documented technique			
Enucleation stratum	APN+ vs APN− within enucleation (n = 85)	10.21 (2.07–50.30)	0.003
Enucleoresection stratum	APN+ vs APN− within enucleoresection (n = 159)	0.99 (0.19–5.12)	>0.99

Analysis	Adjustment/stratum	APN OR (95% CI)	p value
Resection stratum	APN+ vs APN− within resection (n = 157)	1.96 (0.54–7.10)	0.29
Centre-stratified APN OR			
Centre C01	APN+ vs APN− within C01 (n = 182)	2.16 (0.76–6.14)	0.15
Centre C02	APN+ vs APN− within C02 (n = 160)	3.12 (0.99–9.85)	0.06
Centre C03	APN+ vs APN− within C03 (n = 59)	inf (undefined)	0.05
Centre-adjusted	Logistic with centre fixed effects	3.02 (1.44–6.36)	0.004
Sensitivity and robustness			
Full cohort	All 500 patients (incl. benign), univariate	2.87 (1.49–5.51)	0.003
Full cohort adjusted	+ Model 2 covariate set	2.46 (1.22–4.97)	0.01
All-malignant cohort	n = 440 incl chromophobe, univariate	3.10 (1.54–6.25)	0.002
Leave-one-surgeon-out	Univariate OR range (median 2.96)	2.41–4.07	—
Bootstrap (univariate)	2,000 replicates, median OR	3.12 (1.37–7.08)	—
E-value	For unmeasured confounding (point estimate)	5.63	—
Sensitivity G	Model 2 with RENAL substituted for tumour size	2.72 (1.23–6.04)	0.01
Sensitivity H	Model 2 with PADUA substituted for tumour size	2.82 (1.26–6.31)	0.01

Notes (Table S1). All odds ratios (OR) refer to adherent perinephric fat (APN) as the primary exposure and positive surgical margin (PSM) as the binary outcome unless otherwise stated. Confidence intervals are 95% Wald intervals on the log-odds scale. P values reflect two-sided tests of the null. Stratum-specific and centre-specific p values are Fisher exact. Models A–F follow a hierarchical sequence of progressive confounder adjustment. The APN estimate remains stable across all models. In the full-cohort analyses, the 99 cases without an assignable WHO/ISUP grade were coded as not high grade. The Mayo Adhesive Probability score discriminated APN status well in this cohort (AUC 0.84, 95% CI 0.80–0.88 by 1,000-replicate bootstrap). Median follow-up was 44.1 months (IQR 26.5 to 64.2). The E-value [16] for the APN × enucleation interaction (Model 3) is 15.14 for the point estimate (OR 7.83) and 1.36 for the lower 95% CI bound (OR 1.08); the E-value for the adjusted marginal APN OR (Model 2, OR 2.68) is 4.80. An unmeasured confounder would need to have associations of these magnitudes with both APN (or APN-with-enucleation) and PSM to fully explain each estimate. APN, adherent perinephric fat; PSM, positive surgical margin; OR, odds ratio; CI, confidence interval; AUC, area under the curve; IQR, interquartile range; ISUP, International Society of Urological Pathology; PADUA, Preoperative Aspects and Dimensions Used for an Anatomical classification; RENAL, Radius, Endophytic/exophytic properties, Nearness to collecting system, Anterior/posterior, Location.

Table S2 . Firth penalised logistic regression and non-parametric bootstrap distribution details for the pre-specified guideline-based Models 1, 2, and 3 (primary cohort, n = 401, 31 PSM events)

Predictor	Model 1 Firth ∣ OR (95% CI); p	Model 2 Firth ∣ OR (95% CI); p	Model 3 Firth ∣ OR (95% CI); p
High-grade RCC (ISUP ≥3)	2.81 (1.32–5.96); 0.007	2.44 (1.15–5.17); 0.02	2.15 (1.00–4.63); 0.05
Surgeon-documented enucleation	2.37 (1.09–5.14); 0.03	2.00 (0.90–4.42); 0.09	0.75 (0.18–3.04); 0.68
Tumour size (per 1 cm)	1.03 (0.68–1.57); 0.88	1.07 (0.70–1.63); 0.75	1.12 (0.73–1.72); 0.60
cT1b stage (vs cT1a)	2.02 (0.46–8.92); 0.35	2.16 (0.48–9.71); 0.31	2.19 (0.49–9.86); 0.31
Centre C02 (vs C01)	0.96 (0.44–2.08); 0.92	0.95 (0.43–2.07); 0.89	0.94 (0.43–2.07); 0.89
Centre C03 (vs C01)	0.49 (0.13–1.94); 0.31	0.53 (0.13–2.08); 0.36	0.56 (0.14–2.24); 0.41
Adherent perinephric fat	—	2.62 (1.22–5.63);	1.49 (0.57–3.94);

Predictor	Model 1 Firth ∣ OR (95% CI); p	Model 2 Firth ∣ OR (95% CI); p	Model 3 Firth ∣ OR (95% CI); p
(APN)		0.01	0.42
APN × Enucleation interaction	—	—	5.97 (0.96–37.10); 0.06
Penalised log-likelihood	-90.93	-87.19	-84.96
Penalised LR test vs previous model	—	$\chi^2 = 7.48$, df = 1; p = 0.006	$\chi^2 = 4.45$, df = 1; p = 0.03

Bootstrap distribution detail (2,000 percentile replicates)

Estimate	Source model	Bootstrap median	Bootstrap 95% CI	Successful replicates
APN OR	Model 2 (linear, adjusted)	2.60	1.05–6.04	2000/2000
APN × Enucleation interaction OR	Model 3	7.20	1.47–56.07	2000/2000
APN OR within non-enucleation stratum	Model 3 (APN main effect)	1.47	0.42–3.84	2000/2000

Notes (Table S2). This table reports the full Firth penalised logistic regression output and bootstrap distribution details for the pre-specified guideline-based models presented in main-text Table 2. Firth penalisation [15] mitigates small-sample and separation-induced bias in maximum-likelihood estimation; it was implemented by iteratively reweighted modified Newton–Raphson on the Jeffreys-prior-penalised log-likelihood. Confidence intervals for the Firth estimates are Wald intervals on the log-odds scale. The non-parametric percentile bootstrap (2,000 replicates, Firth penalisation applied within each replicate) provides a distribution-free confidence interval not requiring asymptotic approximations and remains stable under quasi-separation in resampled data. Firth penalised logistic regression and standard maximum-likelihood logistic regression yield concordant interpretations for Models 1 and 2. They diverge for the Model 3 interaction, where the Firth Wald p value falls on the other side of the conventional threshold. The APN coefficient in Model 2 is estimated at OR 2.62 (Firth) versus 2.68 (maximum-likelihood), with Firth-bootstrap median 2.60. The APN-by-enucleation interaction term in Model 3 is estimated at OR 5.97 (Firth) versus 7.83 (maximum-likelihood), with Firth-bootstrap median 7.20. Confidence intervals widen at the interaction term, reflecting sparser cell configurations. All three estimators yielded concordant inference for the APN main effect in Model 2 ($p \leq 0.05$). For the APN × enucleation interaction in Model 3, the Firth Wald p value lies at the conventional

significance boundary (p = 0.06; 95% CI 0.96 to 37.10), while the maximum-likelihood point estimate (p = 0.04) and the Firth-bootstrap 95% percentile interval (1.47 to 56.07) exclude the null. The penalised likelihood-ratio test rejects Model 3 against Model 2 ($\chi^2 = 4.45$, df = 1, p = 0.03). * In Model 3, the APN main-effect coefficient gives the APN effect when the enucleation indicator is at its reference level (non-enucleation); the enucleation main-effect coefficient gives the enucleation effect when APN is at its reference level (APN-negative); — = not estimated in this model. APN, adherent perinephric fat; PSM, positive surgical margin; LR, likelihood ratio; CI, confidence interval; ISUP, International Society of Urological Pathology.

Table S3. Surgeon experience confounder test for the APN main effect and the APN × enucleation interaction (primary cohort, n = 401, 31 PSM events)

Estimate	Model 3 (no experience)	Model 4 (+ experience)	Δ relative
APN main effect (within non-enucleation)	OR 1.44 (0.52–4.03); p = 0.48	OR 1.46 (0.52–4.09); p = 0.47	+1%
APN × Enucleation interaction	OR 7.83 (1.08–56.92); p = 0.04	OR 7.79 (1.07–56.79); p = 0.04	<1%
High-grade RCC (ISUP ≥3)	OR 2.19 (0.98–4.88); p = 0.06	OR 2.19 (0.98–4.88); p = 0.06	<1%
Surgeon experience (per 1 case)	— (not in model)	OR 1.00 (0.98–1.01); p = 0.80	—
LR test for experience contribution	—	$\chi^2 = 0.07$, df = 1, p = 0.80	—

Per-surgeon experience and outcomes

Surgeon	Centre	Experience (cases)	Cohort cases	PSM events	PSM rate (%)
S01	C01	120	68	5	7.4
S02	C01	95	61	7	11.5
S03	C01	180	53	4	7.5
S04	C02	140	64	7	10.9

Surgeon	Centre	Experience (cases)	Cohort cases	PSM events	PSM rate (%)
S05	C02	110	53	3	5.7
S06	C02	160	43	3	7.0
S07	C03	60	36	2	5.6
S08	C03	85	23	0	0.0

Notes (Table S3). Surgeon experience was operationalised as the cumulative partial nephrectomy case count per surgeon (pre-cohort experience plus cases performed during the study window; range 60–180, median 115); all eight surgeons exceeded the 30-case experience threshold cited in Carbonara 2022 [8]. Bivariate associations of experience with PSM and with technique selection were examined first, then experience was added to the pre-specified Model 3 as Model 4 to test whether the APN main effect or the APN × enucleation interaction was sensitive to surgeon learning-curve confounding. The bivariate experience–PSM association was null (univariate OR per case 1.00, 95% CI 0.99 to 1.01, p = 0.65), and experience did not predict technique selection in the primary cohort (3×3 chi-square p = 0.55) or in the APN-positive subcohort (3×3 chi-square p = 0.79). Within the APN-positive enucleation subgroup (n = 34, PSM = 10), PSM rates by experience tertile were 21.4% (low, n = 14), 20.0% (mid, n = 10), and 50.0% (high, n = 10); the highest rate falls in the smallest tertile subgroup (5 PSM events). The Model 4 estimates demonstrate that the APN main effect (OR 1.44 → 1.46) and the APN × enucleation interaction (OR 7.83 → 7.79) are insensitive to surgeon experience in this cohort; the experience covariate itself is not a statistically significant PSM predictor (likelihood-ratio test p = 0.80). APN, adherent perinephric fat; LR, likelihood ratio; CI, confidence interval; ISUP, International Society of Urological Pathology.

Table S4 Full baseline characteristics by APN status (variables not displayed in main-text Table 1; primary cohort: ISUP-gradable malignant RCC, n = 401)

Variable	APN− (n = 290)	APN+ (n = 111)	p value
ASA score ≥3	130 (44.8%)	50 (45.0%)	>0.99
Hypertension	123 (42.4%)	53 (47.7%)	0.37
Diabetes	47 (16.2%)	23 (20.7%)	0.30
Baseline eGFR, mL/min	79.7 (66.5–92.7)	79.1 (71.2–90.9)	0.57
PADUA score	7 (6–8)	6 (6–8)	0.50
Papillary RCC	48 (16.6%)	13 (11.7%)	0.28
Sarcomatoid features	6 (2.1%)	1 (0.9%)	0.68
Rhabdoid features	4 (1.4%)	1 (0.9%)	>0.99
Tumour necrosis	27 (9.3%)	13 (11.7%)	0.46
Lymphovascular invasion	22 (7.6%)	6 (5.4%)	0.52
Retroperitoneal	88 (30.3%)	30 (27.0%)	0.54

Variable	APN− (n = 290)	APN+ (n = 111)	p value
approach			
Warm ischaemia time, min	25.0 (21.0–29.0)	23.0 (18.5–30.0)	0.30
Operative time, min	144.0 (127.0–163.0)	141.0 (120.0–158.0)	0.12
Estimated blood loss, mL	217.5 (139.8–273.0)	211.0 (147.0–264.5)	0.61

Notes (Table S4). Continuous variables: median (interquartile range), Mann–Whitney U test. Categorical variables: n (%), Fisher exact or chi-square test. All variables listed are comparable between APN-positive and APN-negative groups in the primary cohort. Variables presented in the main-text Table 1 are not duplicated here. APN, adherent perinephric fat; ASA, American Society of Anaesthesiologists; PADUA, Preoperative Aspects and Dimensions Used for an Anatomical classification; eGFR, estimated glomerular filtration rate; IQR, interquartile range.

Table S5. Interrater agreement between the two assessors for adherent perinephric fat status and for surgeon-documented resection technique (all 500 operative notes)

Adherent perinephric fat	Assessor 2: absent	Assessor 2: present	
Assessor 1: absent	357	13	
Assessor 1: present	18	112	
**Resection technique**	**Assessor 2: enucleation**	**Assessor 2: enucleoresection**	**Assessor 2: resection**
Assessor 1: enucleation	98	13	0
Assessor 1: enucleoresection	5	173	5
Assessor 1: resection	0	20	186

Notes (Table S5). Both assessors scored every operative note independently, blind to margin status and to each other, and the value analysed is the consensus of the two readings. For binary APN status, raw agreement was 93.8% (469/500) and Cohen's kappa 0.84 (95% CI 0.78 to 0.89). For the three-category technique classification, raw agreement was 91.4% (457/500), unweighted kappa 0.87 (95% CI 0.83 to 0.90) and linearly weighted kappa 0.89 (95% CI 0.86 to 0.92). Within the primary analytic cohort (n = 401) the corresponding values were kappa 0.84 (95% CI 0.77 to 0.90) for APN and weighted kappa 0.90 (95% CI 0.86 to 0.93) for technique. Confidence intervals for unweighted kappa are asymptotic; those for weighted kappa are 4,000-replicate bootstrap percentile intervals. APN, adherent perinephric fat; CI, confidence interval.

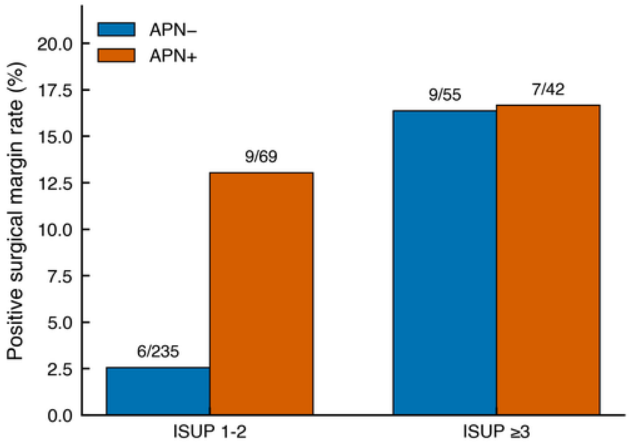

Figure S1. PSM rate by APN status stratified by ISUP nucleolar grade. PSM occurred in 2.6% (6/235) of APN-negative low-grade cases (ISUP 1–2), 13.0% (9/69) of APN-positive low-grade cases, 16.4% (9/55) of APN-negative high-grade cases (ISUP ≥3), and 16.7% (7/42) of APN-positive high-grade cases. The APN × ISUP-grade multiplicative interaction term was inverse (OR 0.18, 95% CI 0.04 to 0.82, p = 0.03), indicating that the APN-associated PSM elevation was attenuated in tumours whose underlying grade already carries an elevated PSM risk.

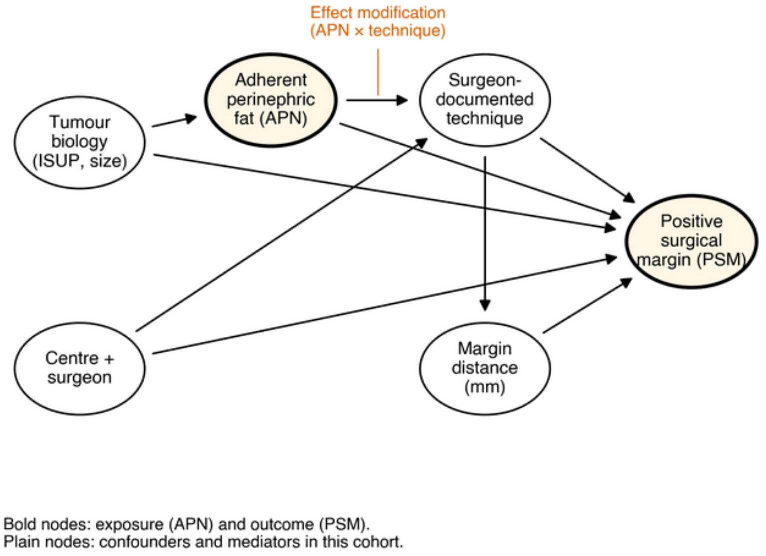

Figure S2. Hypothesised directed acyclic graph for the APN-to-PSM relationship in laparoscopic partial nephrectomy. Bold nodes are the exposure (adherent perinephric fat) and the outcome (positive surgical margin). Tumour biology, entered as ISUP grade and tumour size, is a common cause of both: it influences APN positivity through peritumoral inflammation and margin status through pseudocapsule penetration. Centre and surgeon are common causes of the technique chosen and of margin status. The surgeon observes APN before selecting the dissection plane, so the arrow from APN to surgeon-documented technique represents that choice. Technique therefore lies partly on the pathway from APN to margin status, and part of its influence passes through the achieved margin distance. The analysis treats technique as an effect modifier rather than estimating a mediated effect; the orange connector marks the APN × technique interaction tested in Model 3. Pseudocapsule identifiability is the hypothesised mechanism linking APN to margin failure within the enucleation plane. It was not measured and is therefore not drawn.
